# Proteomics Analysis of Exosomes From Patients With Active Tuberculosis Reveals Infection Profiles and Potential Biomarkers

**DOI:** 10.3389/fmicb.2021.800807

**Published:** 2022-01-06

**Authors:** Min Zhang, Yiping Xie, Shasha Li, Xiaojian Ye, Yibiao Jiang, Lijun Tang, Jianjun Wang

**Affiliations:** ^1^Department of Clinical Laboratory, Affiliated Kunshan Hospital of Jiangsu University, Zhenjiang, China; ^2^Central Laboratory, Affiliated Kunshan Hospital of Jiangsu University, Zhenjiang, China; ^3^Department of Biochemistry and Molecular Biology, School of Life Sciences, Central South University, Changsha, China

**Keywords:** active tuberculosis, exosomes, proteomics, CD36, PBMCs

## Abstract

Although mycobacterial proteins in exosomes from peripheral serum of patients with tuberculosis (TB) have been identified, other exact compositions of exosomes remain unknown. In the present study, a comprehensive proteomics analysis of serum exosomes derived from patients with active TB (ATB) was performed. Exosomes from patients with ATB were characterized using nanoparticle tracking analysis (NTA), transmission electron microscopy (TEM), and western blotting analysis. Then identified protein components were quantified by label-free proteomics and were determined via bioinformatics analysis. A total of 123 differential proteins were identified in ATB serum exosomes and analyzed with Gene Ontology (GO) analysis. Among these proteins heat shock protein70 (HSP70), CD81, major histocompatibility complex-I (MHC-I ) and tumor susceptibility gene101 (TSG101) were present in exosomes of ATB and normal individuals confirmed via western blotting. In addition, among identified exosomal proteins lipopolysaccharide binding protein (LBP) increased significantly, but CD36 and MHC-I decreased significantly in ATB exosomes. Meanwhile, MHC-I was down-expressed in serum and peripheral blood mononuclear cells (PBMCs) of ATB, but interestingly CD36 was down-regulated in serum and up-expressed in PBMCs of ATB patients validated with ELISA and flow cytometry. CD36 was up-regulated by *M. tuberculosis* H37Ra infection in macrophages and suppressed in exosomes from H37Ra infected macrophages detected by western blotting. This study provided a comprehensive description of the exosome proteome in the serum of patients with ATB and revealed certain potential biomarkers associated with TB infection.

## Introduction

*Mycobacterium tuberculosis* (*M. tuberculosis*), the pathogen of tuberculosis (TB), remains an important public health problem around world (Biadglegne et al., 2021). According to a World Health Organization (WHO) report of 2021, there are about 5.8 million new TB cases globally, and nearly 1.5 million patients died from related diseases caused by TB (World Health Organization, 2021). In the past 10 years, a new generation of tools, including genome sequencing and proteomics, have been developed to help us to further understand the disease progression of pathogens in host cells after being processed by the immune system (Sukumar et al., 2014; Cadena et al., 2016). The main techniques for diagnosing active TB are direct microscopic demonstration, culture and polymerase chain reaction (PCR) (Suárez et al., 2019). These traditional diagnostic methods are complicated and time-consuming, which hinders the rapid diagnosis of tuberculosis. Therefore, it is particularly urgent to find diagnostic markers for rapid diagnosis of tuberculosis (Goletti et al., 2016).

Exosomes are 30–150-nm extracellular membrane vesicles and may be a promising research target for the diagnosis and therapy in the infection of tuberculosis, since they are derived from hematopoietic and non-hematopoietic cells and involved in intercellular communication (Wang et al., 2018). Exosomes have important functions in signal transduction, immunomodulation (Alipoor et al., 2016) and the material transportation including lipids, nucleic acids, proteins, and other biochemical substances (Théry, 2011), and they also act as cellular “trash bags” to remove excess intracellular substances (Subramanian et al., 2016), and promote intercellular communication by transporting various molecules from donor cells to recipient cells (Zhang et al., 2018).

Exosomes derived from *M. tuberculosis* infected host cells such as macrophages, nature killer cells trigger different immune responses, such as inflammatory response, antigen presentation, and activate subsequent pathways, highlighting the critical function of exosomes in anti- *M. tuberculosis* immune response (Sun et al., 2021). Exosomes from *M. avium*-infected macrophages induced naïve macrophages to generate proinflammatory factors, such as tumor necrosis factor α (TNFα), inducible nitric oxide synthase (iNOS) and regulated the activation of T cells (Bhatnagar and Schorey, 2007; Bhatnagar et al., 2007). Giri et al. (2010) demonstrated that exosomes derived from *M. avium* infected macrophages contain bacterial pathogenic glycopeptidolipids. Subsequently, various exosome proteomics studies have revealed that exosomes could carry numerous antigens of *M. tuberculosis*. For example, Kruh-Garcia et al. (2014) revealed 41 *M. tuberculosis* proteins within exosomes from macrophages infected by *M. tuberculosis* or *M. tuberculosis* culture filtrate via analysis proteomics. In addition, 20 *M. tuberculosis* proteins in exosomes from the serum of TB patients, such as antigens 85b, Mpt64, GlcB, and BfrB, have been identified (Singh et al., 2011). This evidence suggests that exosomes regulate the immune functions of recipient cells by carrying mycobacterial constituents. These literatures provide valuable information on exosomes in the *M. tuberculosis* infectious process and provide insights into the development of potential exosomal biomarkers for TB diagnosis.

In this study, systematic comparative proteomics profiling analysis of serum exosomes of normal individuals and patients with active TB was performed. Our results revealed different exosomal protein expression panels and indicated that proteins are selectively packaged into exosomes under different physiological conditions. Pathway and functional analyses further indicated the functions of differential proteins in samples from healthy individuals and patients with ATB. These results provide significant evidences on the functions of exosomal proteins during the *M. tuberculosis* infectious process and indicate potential exosomal biomarkers for the diagnosis of TB.

## Materials and Methods

### Ethics Statement

In the present study, human blood samples of normal individuals and patients with ATB were collected in the Clinical Laboratory of Affiliated Kunshan Hospital of Jiangsu University from January 2020 to July 2021. Blood (5 mL/donors) was collected into tubes with heparin anticoagulant and centrifugated at 2,000 × g for 10 min to obtain serum the serum was stored at –80°C for further analysis. Blood (5 mL/donors) in tubes with EDTA anticoagulant was centrifugated at 2,000 × g, for 10 min through Ficoll Hypaque (5 mL, Thermo Fisher Scientific, United States) density gradient centrifugation, then mononuclear cells that retaining the interface between plasma and stratification fluid were acquired. Total 55 ATB patients were diagnosed according to combined clinical criteria from the WHO (World Health Organization et al., 2001). A total of 45 non-*M. tuberculosis* infected individuals with the results of negative tuberculin skin tests were included in the normal group as negative control (NC). There was no significant differences in age, sex ratio, body mass index (BMI), and smoking history among ATB patients and NC groups (*P* > 0.05) (Table 1).

**TABLE 1:** Characteristics of patients with ATB and NC groups.

Participant characteristics	Severe ATB	Mild ATB	NC	*P*-value (SATB vs. MATB)	*P*-value (SATB vs. NC)
Diagnosis (n)	33	22	45		
Age, years	39.26 ± 14.35	41.83 ± 12.64	40.65 ± 10.86	0.121	0.104
Male (n)	19	13	22	0.514	0.629
Female (n)	14	9	23		
BMI	21.269 ± 2.28	22.28 ± 2.32	22.54 ± 2.15	0.078	0.062
Smoking history, n (%)	13 (39.39)	10 (45.45)	14 (31.11)	0.654	0.861

*Age and BMI are presented as Mean ± SD. ATB, active tuberculosis; BMI, body mass index; n, number of subjects; NC, negative control; SATB, severe active tuberculosis; MATB, mild active tuberculosis.*

This study was ethically approved by the Ethics Committee of Affiliated Kunshan Hospital of Jiangsu University (Suzhou, China, approval no. IEC-C-007-A07-V3.0). The experiments were performed in accordance with the Declaration of Helsinki Principles, and all ATB patients and normal individuals provided their written informed consent to participate in the present study.

### Cell Culture and H37Ra Infection

Inactivated *M. tuberculosis* H37Ra was provided by the Center for Chinese Disease Prevention and Control (Beijing, China). THP-1 cells were purchased from The Cell Bank of Type Culture Collection of Chinese Academy of Sciencess (Shanghai, China). THP-1 cells were cultured in wells or flasks with RPMI-1640 containing 10% exosome-depleted FBS, 100 U/mL penicillin and 0.1 mg/mL streptomycin at 37°C with 5% CO_2_. THP-1 cells were induced into macrophages using Phorbol 12-myristate 13-acetate (0.1 mM) for 24 h.

Macrophages were infected with H37Ra at a multiplicity of infection of H37Ra: cell = 100:1 for 24 h. Subsequently, the infected macrophages were washed with PBS three times for next experiments.

### Cell Transfection

Small interfering RNA (siRNA) of CD36 (50 nM; Shanghai GenePharma Co., Ltd., Shanghai, China) was transfected into macrophages grown in 6-well plates at 75% confluence using Lipofectamine 3000^®^ reagent (Invitrogen; Thermo Fisher Scientific, Inc., Waltham, MA, United States) at 37°C for 24 h according to the manufacturer’s protocols. CD36-siRNA (50 pM) was added into serum-free diluent and mixed to siRNA diluent (25 μL). Lipofectamine 3000^®^ reagent (1 μL) was added into serum-free diluent (24 μL) and mixed to Lipofectamine 3,000 diluent at room temperature for 5 min. Then, Lipofectamine 3,000 diluent and RNA diluent were mixed together at room temperature for 15 min to form the transfection complex. The transfection complex (50 μL) was added into 2 × 10^5^ macrophages with whole medium (0.45 mL) and mixed gently, and transfection cells were cultured for 24 h after transfection. The CD36 siRNA sequence was 5′-GGAAUCCCUGUGUAUAGAUTTAUCUAUACACAGGGAU UCCTT-3′ and the scrambled siRNA sequence was 5′-UUCU CCGAACGUGUCACGUTTACGUGACACGUUCGGAGAA TT-3′.

### Isolation and Identification of Exosomes

Exosomes from serum and cell culture supernatants were isolated with 0.9% sucrose solution via differential centrifugation. Briefly, all serum and cell supernatants were centrifuged at 300 × g for 10 min to remove cells, 3,000 × g for 15 min to remove cell debris, 10,000 × g for 60 min to remove microvesicles and 100,000 × g for 2 h to acquired exosomes, every steps followed by two washes with PBS buffer and all performed at 4°C. The purified exosomes were identified by with NTA, TEM, and western blotting. The total protein concentration of exosomes was measured with BCA Assay (Thermo Fisher Scientific, Inc., Waltham, MA, United States). Before the experiment, all isolated exosomes were stored at −80°C.

### Nanoparticle Size Analysis

The isolated exosomes were suspended in PBS solution (100 μL). After fully shaking and mixing, exosomes (10 μL) were diluted with ultrapure water to 1 mL, and injected into the Nano-sight S300 nano-particle size analyzer. The particle size distribution and concentration were measured according to the standard process at 4°C. Each exosomes sample was analyzed three times using Nano-sight NTA (V3.2) software.

### Transmission Electron Microscopy Analysis

Exosomes were re-suspended in PBS (100 μL). A total of exosome diluted solution (10 μL) was dropped on the copper wire at 25°C for 5 min, and washed with ultrapure water three times. After air drying, 10 μL phosphotungstic acid solution was added on copper mesh coloring for 2 min for air drying at room temperature. Test samples were analyzed with Hitachi H-600 transmission electron microscope (Hitachi, Ltd., Tokyo, Japan) to observe the morphology of exosomes.

### Flow Cytometry Analysis

Exosomes (30 μg) were added to beads (10 μL) and attached to aldehyde/sulfate latex beads (Invitrogen; Thermo Fisher Scientific, Inc., United States) at 25°C for 15 min. Subsequently, the aforementioned treated exosomes were diluted with PBS and incubated at 4°C overnight. Exosome-bound beads were washed with PBS containing 0.5% bovine serum albumin (BSA) three times. After centrifugation, the bead pellet was resuspended in PBS (0.5 mL) containing 0.5% BSA. Coated beads were incubated with CD9, CD63, and CD81 antibodies (Biolegend, Inc., San Diego, CA, United States) at 4°C for 30 min.

Fresh peripheral blood (100 μL) from health individuals and ATB patients was incubated with MHC-I, CD14, CD36, and CD69 antibodies (respectively, 5 μL) (Biolegend, Inc., San Diego, CA, United States) for 15 min. After that, red blood cell lysate was added and cell precipitation was obtained by 300 × g centrifugation, and resuspend with PBS (500 μL) for next step. Analysis were performed on a BD FACSCanto™ II flow cytometer (Becton, Dickinson and Company, United States). Fluorescence intensity values were obtained, and mean fluorescence intensities were used for statistical analysis.

### Protein Extraction and Digestion

In the present study, the exosome samples were divided into two groups: One group comprising exosomes purified from the serum of normal individuals and another group comprising exosomes purified from the serum of patients with ATB, with three replicates per group.

Exosomes were extracted with SDT buffer (4% SDS, 100 mM Tris-HCl, 1 mM DTT, pH 7.6), and the protein content was monitored with a BCA assay kit (Bio-Rad, United States). Then the exosomal proteins were digested by trypsin as per the filter-assisted sample preparation from Matthias Mann. The resulting peptides were desalted on Empore SPE Cartridges C18 (standard density, bed I.D. 7 mm, volume 3 mL, Sigma), vacuum-centrifuged and reconstructed in 40 μL of 0.1% (v/v) formic acid. Proteins (200 μg) were loaded into 30 μL of another SDT buffer (4% SDS, 100 mM DTT, 150 mM Tris-HCl pH 8.0). The detergent DTT and other low-molecular-weight substances were repeatedly ultrafiltered with a UA buffer (8 M Urea, 150 mM Tris-HCl, pH 8.0) (Microcon units, 10 kD). Then the reduced cysteine residues were blocked by adding 100 μL of iodoacetamide (100 mM IAA in the UA buffer) and cultured for 30 min in the dark. The filters were cleaned first with 100 μL of the UA buffer three times and then with 100 μL of 25 mM NH_4_HCO_3_ twice. Finally, the protein suspensions were digested with 4 μg of trypsin (Promega Corporation, United States) in 40 μL of the NH4HCO3 buffer at 37°C overnight, and the final peptides were harvested as a filtrate.

The UV spectral density at 280 nm of the peptides were measured using an extinction coefficient of 1.1 of a 0.1% (g/l) solution that was computed as per the frequency of tryptophan and tyrosine in vertebrate proteins.

### LC-MS/MS Analysis

LC-MS/MS was run on a Q-Exactive MS meter (Thermo Fisher Scientific, United States) equipped with Easy nLC (Thermo Fisher Scientific, United States) for 120 min. The peptides were placed onto a PepMap100 reversed trap column (Thermo Fisher Scientific Acclaim, 100 μm*2 cm, nanoViper C18) linked to a C18-reversed analytical column (Thermo Fisher Scientific Easy Column, 10 cm long, 75 μm I.D., 3 μm resin) in buffer A (0.1% Formic acid) and isolated with a linear gradient of buffer B (84% acetonitrile, 0.1% Formic acid) at a flow speed of 300 nl/min managed by IntelliFlow. MS data in the positive ion mode were collected using a data-based top10 approach to dynamically select the richest precursor ions from the scan range of 300–1,800 m/z for HCD decomposition. Maximum injection time, automatic gain control target and dynamic exclusion duration were set at 10 ms, 3e6 and 40.0 s, respectively. Survey scanning resolution, HCD spectral resolution, and isolation width were 70,000 at m/z 200, 17,500 at m/z 200, and 2 m/z, respectively. Normalized collision energy was 30 eV, and the under fill ratio, referring to the lowest percent of the target value to be met at longest fill time, was 0.1%. The peptide recognition mode was chosen here.

The raw MS data for each sample were integrated and searched on MaxQuant 1.5.3.17 for identification and quantification.

### Bioinformatic Analysis

Hierarchical clustering analysis was performed by Cluster 3.0.^1^ In addition to tree diagrams, heat maps and volcano plot are usually presented as visual aids. The subcellular localization of exosomal proteins was predicted with CELLO subcellular localization predictive system.^2^

Based on the online Kyoto Encyclopedia of Genes and Genomes (KEGG) database^3^ (Huang et al., 2009), we conducted a blast of differential exosomal proteins to retrieve their KEGG orthology identification, and then mapped to the path in KEGG.

The enrichment analysis is based on Fisher’s exact test, using the entire quantitative protein as the background data set. A *P*-value < 0.05 was set to distinguish statistically significant enrichment results. The functional interactions between proteins can be plotted to illustrate the molecular mechanisms and signaling pathways of cellular processing. The protein–protein interaction (PPI) of exosomal proteins was analyzed with Retrieval of Interacting Genes (STRING)^4^ database (Szklarczyk et al., 2015), and subsequently was visualized using Cytoscape^5^ (Shannon et al., 2003).

### Enzyme-Linked Immunosorbent Assay

MHC-I, CD36, and lipopolysaccharide binding protein (LBP) levels in the serum of 40 normal individuals and 40 patients with ATB were measured by using ELISA. After the serum was treated by ultrasound to broke serum exosomes with intermittent ultrasonic treatment, the concentrations of MHC-I, CD36, and LBP in serum were examined according to the reagent operating instructions (Invitrogen; Thermo Fisher Scientific, Inc., Waltham, MA, United States).

Briefly, Standards or serum samples (100 μL) were added in appropriate wells incubated for 0.5 h at 37°C, and then wells were washed 3 times with wash solution. Biotinylated antibodies (100 μL) of MHC-I, CD36, and LBP were added to each well incubated for 1 h at 37°C, were washed 3 times with wash solution. Streptavidin solution (100 μL) was added in each well incubated for 45 min at 37°C and then wells were washed 3 times with wash solution. MB substrate reagent (100 μL) was added to each well incubated for 30 min at 37°C in the dark and finally, stop solution (50 μL) was added to each well. Absorbance was detected at 450 nm using a Spectra Max 190 ELISA reader (Molecular Devices, LLC, Sunnyvale, CA, United States) and the concentrations were quantified against the standard curve.

### Western Blotting

Total proteins were isolated from fresh macrophages cells (5.5 × 10^6^) or frozen exosomes (100 μg) were lysed using RIPA peptide lysis buffer (Beyotime Institute of Biotechnology, Haimen, China). Proteins (30 μg) were loaded on 10% SDS-PAGE gels, electrophoresed, and then transferred to PVDF membranes (MilliporeSigma, Burlington, MA, United States). Subsequently, the membranes were incubated with primary antibodies against CD81 (at 1:2,000 dilution, ProteinTech Group, Inc., Chicago, United States), HSP70 (at 1:3,400 dilution, Biolegend, Inc., San Diego, CA, United States), LBP (at 1:2,400 dilution, ProteinTech Group, Inc.), CD36 (at 1:2,000 dilution, Biolegend, Inc.), MHC-I (at 1:3,000 dilution, Biolegend, Inc.) and TSG101 (at 1:2,000 dilution, ProteinTech Group, Inc.) at 4°C overnight. Then, PVDF membranes were washed three times with TBS-T under shaking. Finally, the membranes were incubated with secondary antibody solution (mouse anti-rabbit IgG-HRP at a 1:10,000) for 60 min at room temperature with gentle shaking, and washed with TBS-T under shaking three times again. Protein bands were imaged and analyzed using the Chemiluminescent Substrate System (Thermo Fisher Scientific, Inc., Waltham, MA, United States).

### Statistical Analysis

All data in this study are presented as the mean ± standard error of the mean. Statistical analysis was performed with GraphPad Prism 5 (GraphPad Software Inc., San Diego, CA, United States). Differences among groups were analyzed using one-way ANOVA or a paired Student’s *t*-test. The statistical results are considered to be statistically significant differences (*P* < 0.05).

## Results

### Characterization of Serum Exosomes From Patients With Active TB

**FIGURE 1:**
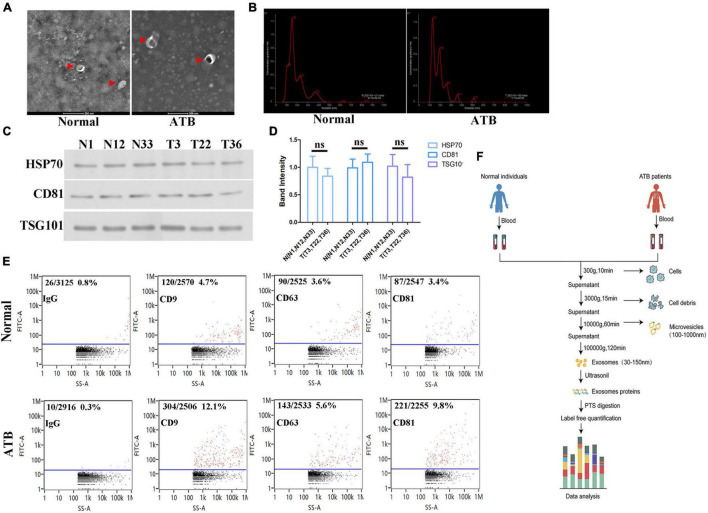
Biological characterization of exosomes isolated from serum. **(A)** Transmission electron micrographs of isolated exosomes from the serum of normal individuals and patients with ATB. Representative image of exosomes at a magnification of × 200. **(B)** Size distribution of exosomes was determined by using NTA. Particle size (150 nm) and concentrations (2.01E10 particles/mL). **(C,D)** HSP70 and CD81 in exosomes from normal individuals (N#1, N#12, and N #33) and patients with ATB (T#3, T#22, and T #36) were quantified via western blotting. TSG1-101 was used as a control. **(E)** Exosomal marker proteins CD9, CD63, and CD81 on exosomes were detected by using flow cytometry. **(F)** Schematic diagram of separation and proteomic analysis of serum exosomes.

The exosomes were typical cup-shaped vesicles as observed by TEM, and no differences in shape between the exosomes from the serum of normal individuals and patients with ATB were observed (Figure 1A). NTA analysis revealed that the particle size distribution was within 30–150 nm. Furthermore, the diameter of most particles was 150 nm, indicating that these particles were exosomes (Figure 1B). Western blot analysis further confirmed the presence of surface markers of exosomes, such as HSP-70, CD81, and TSG101 (Figures 1C,D). The flow cytometry results suggested that the exosomes were positive for CD9, CD63, and CD81, and negative for immunoglobulin G (Figure 1E). In conclusion, pure exosomes were obtained by differential ultracentrifugation, which laid the foundation for subsequent proteomics (Figure 1F).

### Proteomics Analysis of Exosomes Derived From the Serum of Patients With Active TB

**FIGURE 2:**
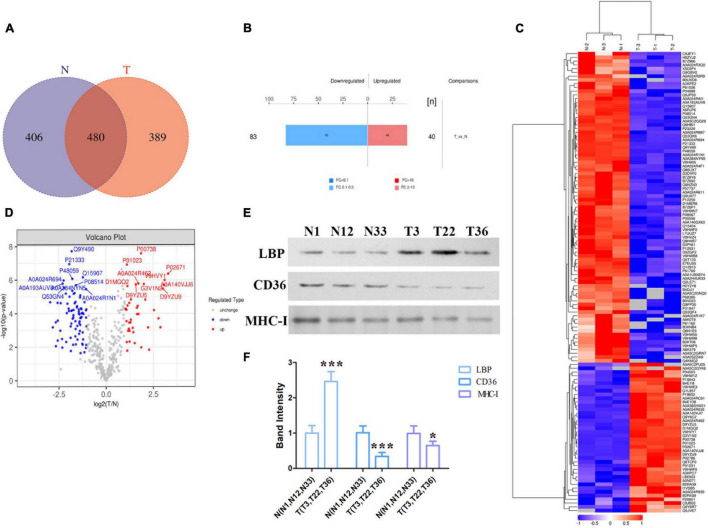
Bioinformatics analysis of exosome differential proteins. **(A)** Venn diagram illustrating overlapping proteins in the two independent MS experiments (T vs. N). **(B)** Histogram of quantitative difference results of two groups of MS experimental proteins, including 40 up-regulated proteins and 83 down-regulated proteins. **(C)** Heat map of the protein levels of shared proteins among exosomes released from the serum of normal individuals and patients with ATB. **(D)** Volcano diagram of differential expression of exosome proteins between the two groups (T vs. N). **(E,F)** Differential proteins (LBP, CD36, and MHC-I ) in exosomes of the two groups (T vs. N) were quantified via western blotting. *p < 0.05, ***p < 0.001.

To determine the exosomes protein profile in the serum of patients with ATB, total exosomal proteins were identified through label-free quantitative proteomics analysis by Applied Protein Technology Co., Ltd. Protein database searching of tandem mass spectrometry (MS/MS) data resulted in the identification of 886 exosomal proteins in the serum of patients with ATB and 869 exosomal proteins in the serum of normal volunteers. Furthermore, 480 exosomal proteins were identified in both experimental groups, as shown in the Venn diagram (Figure 2A). Bioinformatics analysis of proteomics shows that there were 123 differentially expressed proteins including 40 up-regulated proteins and 83 down-regulated proteins were indicated by histogram, cluster analysis (Figure 2B), heat map (Figure 2C), and volcano plot (Figure 2D). Furthermore, 13 up-expressed and 30 down-expressed proteins are listed in Tables 2, 3, respectively. Interestingly, LBP, CD36, and MHC-I molecules were confirmed by western blotting (Figures 2E,F).

**TABLE 2:** List of 13 up-regulated proteins with significant interesting in exosomes from ATB patients.

Number	Protein	Protein name	Gene	Proteins	Peptides	Unique peptides	Coverage	Mol/Weigh
1	P02671	Fibrinogen alpha chain	FGA	10	59	59	63.4	94.972
2	G3V1N2	HCG1745306, isoform CRA_a	HBA2	1	9	1	94.5	11.948
3	A0N071	Delta globin	HBD	17	14	3	79.6	16.055
4	A0A024R035	Complement component C9	C9	3	25	25	32.7	63.203
5	V9HWE3	Carbonic anhydrase	HEL-S-11	15	15	15	76.6	28.87
6	Q9Y6C2	EMILIN-1	EMILIN1	2	27	27	37.8	106.69
7	A0A024RC61	Aminopeptidase	ANPEP	12	25	25	28.2	109.54
**8**	**Q8TCF0**	**Lipopolysaccharide-binding protein**	**LBP**	**2**	**10**	**10**	**19.7**	**52.933**
9	P02786	Transferrin receptor protein 1	TFRC	6	33	1	46.4	84.87
10	P01023	Alpha-2-macroglobulin;α-2	A2M	6	84	72	68	163.29
11	C9JB55	Serotransferrin	TF	1	7	1	82.7	7.99
12	P19652	Alpha-1-acid glycoprotein 2	ORM2	1	8	5	44.3	23.602
13	Q1L857	Ceruloplasmin	N/A	12	47	47	52.3	115.47

*Bold values are proteins that were analyzed in this study.*

**TABLE 3:** List of 30 down-expressed proteins with significant interesting in exosomes from ATB patients.

Number	Protein	Protein name	Gene	Proteins	Peptides	Unique peptides	Coverage	Mol/Weigh
1	A3KPE2	Apolipoprotein C-III	APOC3	3	4	4	48.5	10.852
2	V9HVZ4	Glyceraldehyde-3-phosphate dehydrogenase	HEL-S-162eP	10	15	15	58.5	36.053
3	B0UXD8	HLA-DRA	HLA-DRA	86	4	4	22.4	28.607
4	P04899	Guanine nucleotide-binding protein G(i) subunit alpha-2	GNAI2	42	18	14	58.6	40.45
**5**	**E7EU05**	**Glycoprotein IIIb**	**CD36**	**30**	**13**	**13**	**30.9**	**52.062**
6	P23229	Integrin alpha-6	ITGA6	3	38	31	43.7	126.6
7	A0A024R4F1	2-phospho-D-glycerate hydro-lyase	HEL-S-17	23	17	15	47.7	47.168
**8**	**G8GBV0**	**MHC class I antigen**	**HLA-A**	**1,288**	**17**	**0**	**61.9**	**31.612**
9	P07737	Profilin-1	PFN1	4	7	7	49.3	15.054
10	L7UUZ7	Integrin beta	ITGB3	16	36	4	49.6	86.998
11	Q5JP53	Tubulin beta chain	TUBB	47	17	3	52.6	47.766
12	V9HWF0	Integrin-linked protein kinase	HEL-S-28	7	14	14	41.2	51.419
13	A0A024R611	Coronin	CORO1A	11	14	14	29.7	51.026
14	V9HWN7	Fructose-bisphosphate aldolase	HEL-S-87p	16	20	17	71.7	39.42
15	G9FP35	Guanine nucleotide binding protein	GNAQ	10	12	11	37.3	42.111
16	D3DVF0	Junctional adhesion molecule 1	F11R	6	10	10	36.1	32.227
17	Q9NZN3	EH domain-containing protein 3	EHD3	4	14	6	29.2	60.886
18	A0A024R3Q0	ADP-ribosylation factor 1, isoform CRA_a	ARF1	15	7	4	44.2	20.697
19	V9HWF5	Peptidyl-prolyl cis-trans isomerase	HEL-S-69p	18	9	9	59.4	18.012
20	B0V023	C6orf25	C6orf25	3	7	2	42.2	25.003
21	X6RJP6	Transgelin-2	TAGLN2	4	9	9	55.1	21.086
22	Q12913	Receptor-type tyrosine-protein phosphatase eta	PTPRJ	6	27	27	23.9	145.94
23	P08567	Pleckstrin	PLEK	2	10	10	34	40.124
24	P48059	LIM and senescent cell antigen-like-containing domain protein 1	LIMS1	17	16	14	56	37.251
25	Q86UX7	Fermitin family homolog 3	FERMT3	9	43	43	66.3	75.952
26	Q9Y490	Talin-1	TLN1	7	131	131	61.9	269.76
27	P21333	Filamin-A	FLNA	20	141	111	67.9	280.74
28	V9HWI5	Cofilin, non-muscle isoform	HEL-S-15	11	15	15	74.1	18.502
29	P61160	Actin-related protein 2	ACTR2	7	8	8	24.9	44.76
30	A8K0T9	F-actin-capping protein subunit alpha	N/A	2	9	7	48.6	32.908

*Bold values are proteins that were analyzed in this study.*

### Bioinformatics Analyses of Exosomes From Patients With Active TB

**FIGURE 3:**
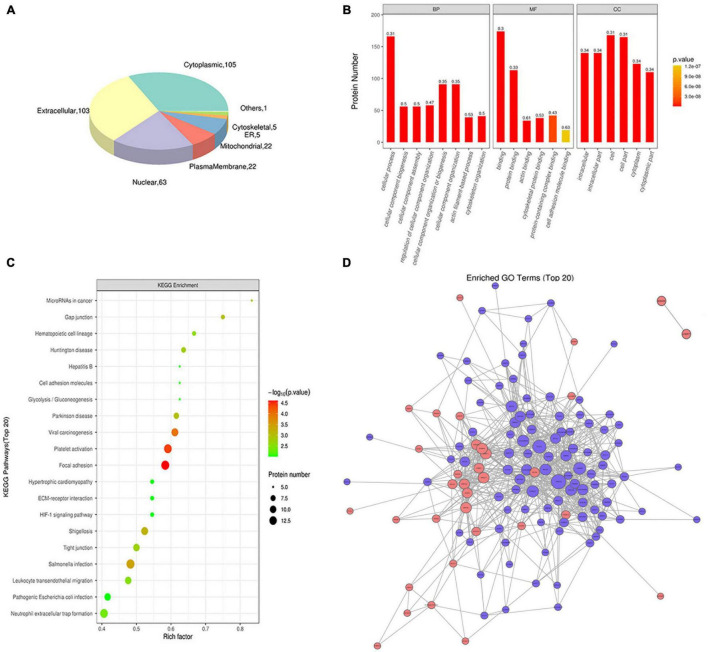
Functional analysis of exosomes differential proteins. **(A)** Subcellular localization pie chart of differentially expressed proteins in two groups of exosomes (T vs. N). **(B)** GO enrichment map of exosomal differential proteins, including BP, MF, and CC. **(C)** Bubble diagram of KEGG pathway enrichment of exosomal differential proteins. **(D)** Interaction network of exosomes differentially expressed proteins.

Subcellular localization analysis of exosomes differential proteins revealed that the identified proteins were enriched in the extracellular region (103 proteins), cytoplasm region (105 proteins), nuclear region (63 proteins), membrane region (22 proteins), mitochondrial region (22 proteins), and 11 other proteins (Figure 3A).

The Gene Ontology (GO) analysis grouped the results according to biological processes (BP), cellular components (CC), and molecular functions (MF). In terms of biological processes, exosome proteins were mainly involved in cellular process, biological regulation or process, response stimulus, and other functions (Figure 3B). As for molecular functions, exosomal proteins were enriched in molecule with molecular binding and catalytic activities (Figure 3B). In terms of the cellular components, exosomal proteins were enriched in cell, intracellular and cytoplasm region (Figure 3B). The KEGG pathway analysis revealed that exosomal proteins were enriched in focal adhesion, platelet activation, viral carcinogenesis etc. (Figure 3C). Based on intact database, the network diagram of differential protein interaction in exosomes was obtained using Cytoscape software, and the interaction relationship of differential proteins were clearly displayed (Figure 3D).

The analyses indicated that these identified exosomal proteins act as a tool for exosomes research and emphasize the need for further experimental evaluation of the functions of the identified exosomal proteins in physiology and pathophysiology of patients with ATB.

### Major Histocompatibility Complex-I, CD36, and Lipopolysaccharide Binding Protein Levels in Serum Confirmed by Enzyme-Linked Immunosorbent Assay

**FIGURE 4:**
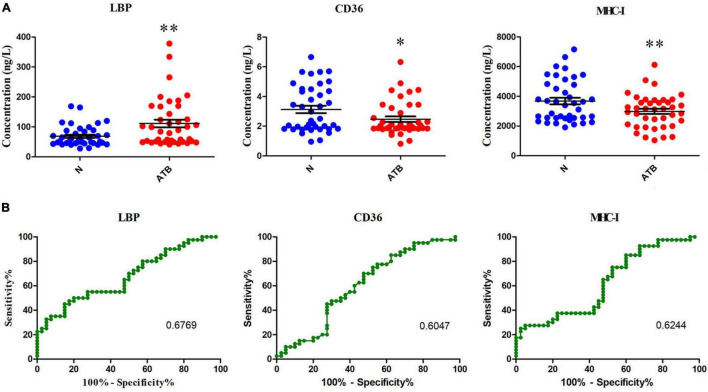
MHC-I, CD36, and LBP expression in the serum of normal individuals and patients with ATB detected using ELISA kits. **(A)** Concentrations of MHC-I, CD36, and LBP in the serum were analyzed. **(B)** MHC-I, CD36, and LBP in the serum to distinguish patients with ATB infection from healthy controls. The AUC values of MHC-I, CD36, and LBP were 62.44, 60.47, and 67.69%, indicating that they can be used to distinguish patients with ATB infection from healthy controls (ROC, receiver operating characteristic). *p < 0.05, **p < 0.01.

The differential exosomal proteins MHC-I, CD36, and LBP in the serum were confirmed in normal individuals and patients with ATB by using ELISA. The expression levels of MHC-I and CD36 were decreased, but LBP expression was markedly increased in patients with ATB compared with normal controls (Figure 4A). In addition, the diagnostic performances of these three protein were analyzed using receiver operating characteristic (ROC) curves (Figure 4B). The area under the curve values of MHC-I, CD36, and LBP were 62.44, 60.47, and 67.69%, respectively, revealing that MHC-I, CD36, and LBP may be potential biomarkers for the diagnosis of ATB infection.

### Surface Markers on Peripheral Blood Mononuclear Cells Determined by Flow Cytometry

**FIGURE 5:**
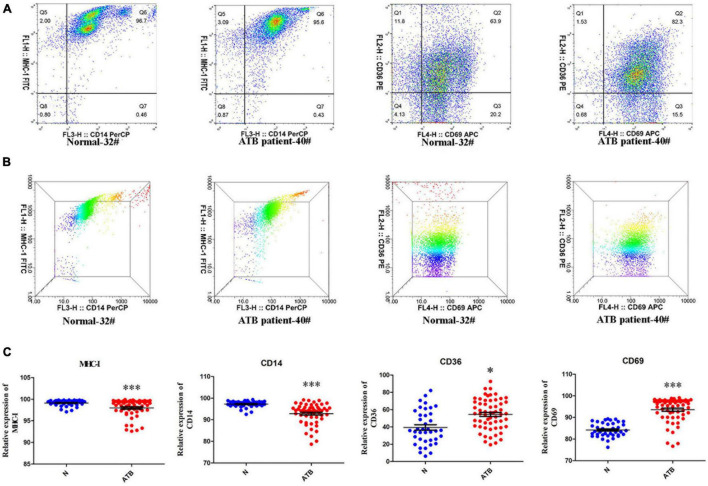
MHC-I, CD36, CD69, and CD14 expression in the PBMCs of normal individuals and patients with ATB. **(A,B)** Scatter diagram and three dimensional results of MHC-I, CD36, CD69, and CD14 expressions in PBMCs of normal individual-32# and ATB patient-40# were detected by flow cytometry and analyzed using flowjo10 software. **(C)** Scatter statistical results for MHC-I, CD36, CD69, and CD14 in 40 normal individuals and 55 ATB patients were quantified using GraphPad Prism 5 software. *p < 0.05, ***p < 0.001.

MHC-I, CD14, CD36, and CD69 expression on PBMCs of 40 healthy individuals and 55 patients with TB was examined via flow cytometry (Figures 5A,B). The experimental results demonstrated that MHC-I and CD14 were markedly down-regulated, however, CD36 and CD69 were markedly up-regulated on PBMCs of patients with ATB compared with healthy individuals (Figure 5C). Interestingly, these results of the expression levels of MHC-I and CD36 were consistent with the results of exosome proteomics and ELISA for serum analyses.

### Expression Levels of CD36 Regulated by H37Ra

**FIGURE 6:**
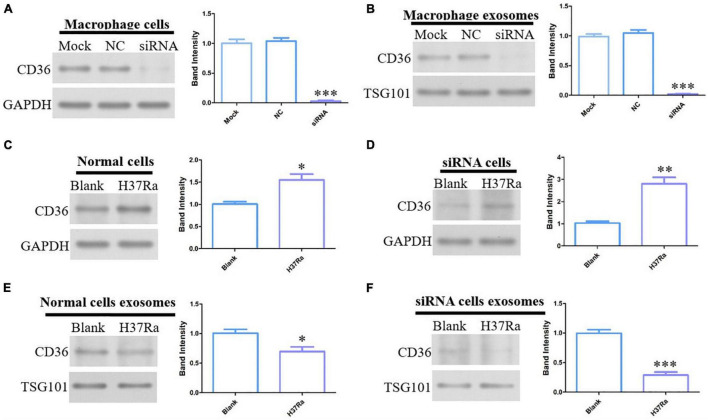
CD36 expression in H37Ra-infected macrophages or exosomes determined by western blotting. **(A)** CD36 in macrophages was knocked down using siRNA. **(B)** CD36 expression was analyzed in exosomes derived from CD36-knockdown macrophages. **(C,D)** CD36 expression was promoted in H37Ra-infected macrophages and CD36-knockdown macrophages. **(E,F)** H37Ra infection suppressed the expression levels of CD36 in macrophages and CD36-knockdown macrophages. *p < 0.05, **p < 0.01, ***p < 0.001.

In view of the expression levels of CD36 on exosomes proteomics, serum ELISA and peripheral blood mononuclear cells, CD36 was selected as the target of our in-depth study. To explore the effect of H37Ra infection on CD36 in macrophages, CD36 was knocked down in macrophages. CD36 protein expression was markedly decreased on treated macrophages and exosomes derived from these compared with the expression levels in the NC or mock groups (Figures 6A,B). Additionally, H37Ra infection promoted CD36 expression on macrophages (Figures 6C,D) but CD36 expression was down-regulated on exosomes from normal macrophages and siRNA-transfected macrophages (Figures 6E,F). These findings revealed that H37Ra infection regulated CD36 expression and transmission via exosomes.

## Discussion

Accumulating studies have reported that a number of mycobacterial proteins in exosomes have been identified using MS (Dobos et al., 2011; Wang et al., 2019). Furthermore, these exosomal proteins are immunogenic and function in intracellular communication, for example, by stimulating the T cell immune response (Giri and Schorey, 2008; Singh et al., 2011). Interestingly, several markers, including HSP70 and tetraspanins (CD9, CD63, and CD81), are conserved on exosomes derived from various cells (Anand et al., 2010; Wang et al., 2014; Kowal et al., 2017). These specific differentially expressed exosome molecules are potential molecular markers for the diagnosis of *M. tuberculosis* infection. In the present study, exosomes isolated from the serum of patients with ATB and healthy individuals were round and 30–150 nm in diameter. The characteristic proteins CD9, CD63, CD81, HSP70, and TSG-101 were all expressed on exosomes from ATB patients. According to the size and marker molecule expression of these vesicles, our experiments have obtained high-purity exosomes, which are essential for downstream proteomics analysis.

An inspection of the proteome dataset for exosomes derived from the serum of patients with ATB and healthy individuals by liquid chromatography with MS/MS revealed the presence of various proteins common to exosomes from various origins, including HSPs, integrins, cytoskeletal proteins, and enzymes, which may be associated with functions of structure, biogenesis, and trafficking of exosomes. 480 overlapping exosomal proteins from the serum of normal volunteers and patients with ATB were revealed in the Venn diagram by comparing the gene symbols of the identified exosomal proteins from the two exosome groups using ExoCarta. However, there were 123 proteins with significant differences, including 40 up-regulated proteins (such as: complement component C9, carbonic anhydrase, aminopeptidase, lipopolysaccharide-binding protein, transferrin receptor protein 1, alpha-1-acid glycoprotein 2, etc.) and 83 down-regulated proteins (for example: glycoprotein IIIb, MHC class I antigen, profilin-1, junctional adhesion molecule 1, cofilin, actin-related protein 2, etc.). These differential exosomal proteins were revealed by the quantitative difference histogram, the cluster analysis heat map and the volcanic map. At the same time, we also found that there are 115 no differentially expressed exosomal proteins (for instance: afamin, alpha-2-HS-glycoprotein, vitamin D-binding protein, catalase, etc.) in the serum of ATB patients.

To obtain systematic insights into the proteome profiles of exosomes, the present study examined the differentially expressed proteins by using bioinformatics analysis, including GO, KEGG, and protein-protein interaction analysis. The most enriched pathways were cellular process and biological regulation. In addition, among these differential proteins, LBP, CD36, and MHC-I attracted our interest and these three molecules are closely related to the infection of tuberculosis, especially CD36. Recent report show that CD36 elevated in exosomes derived from bladder cancer cells by proteomics analysis (Welton et al., 2010). So they were selected to be as a research target and confirmed further by western blotting. The results demonstrated that LBP expression was increased and the expression levels of CD36 and MHC-I were decreased in exosomes from the serum of patients with ATB. In addition, LBP was increased, but CD36 and MHC-I were decreased in the serum of ATB patients determined by ELISA analysis. At the same time, the ROC analysis of LBP, CD36, and MHC-I indicated that they possessed the value of diagnostic biomarker for ATB.

LBP is important for the recognition of lipopolysaccharides by the host and thereby for the induction of an adequate innate immune response to Gram-negative bacteria (Liu et al., 2021). However, Branger et al. (2005) reported that endogenous LBP is not important for host defense against virulent *M. tuberculosis* or avirulent *M. smegmatis*. Furthermore, it was revealed that LBP is markedly up-regulated in the serum of patients with TB. Although there is almost no literature regarding LBP in *M. tuberculosis* infection, its abnormal change in the serum of patients with ATB suggests that it may be associated with the infection of *M. tuberculosis*, but its roles in the infection process has not yet been discovered.

MHC-I-restricted cytotoxic T lymphocytes are known to serve an important role in the control of *M. tuberculosis* infection (Wang et al., 2011). At the same time, MHC-I could present antigens of *M. bovis BCG*, which are associated with the biosynthesis and transport of lipids and are located at the cell membrane (Bettencourt et al., 2020). In the present study, it was revealed that MHC-I expression was markedly down-regulated in the serum and PBMCs of patients with TB. Low expression levels of MHC-I *in vivo* lead to weakening of the presentation ability of TB antigens and ineffective resistance to infection with *M. tuberculosis*.

Three cell surface molecules (CD14, CD69, and CD36) closely associated with ATB infection were also investigated. Our study indicated that the expression levels of CD14 were down-regulated, but the expression levels of CD69 and CD36 were enhanced in PBMCs of patients with ATB. CD36 is a pattern recognition receptor expressed on macrophages and could recognize *M. tuberculosis* (Silverstein and Febbraio, 2009; Lao et al., 2017). It has been demonstrated that macrophages deficient in CD36 restrict the growth of multiple mycobacterial species *in vitro*, and CD36 deficiency confers resistance to mycobacterial infection in mice (Hawkes et al., 2010). Surprisingly, the present results revealed that CD36 was down-regulated in the serum of patients with ATB but was up-regulated in PBMCs. It is not clear why these opposite results for CD36 expression in the serum and PBMCs were observed. CD36 mediates surfactant lipid uptake of macrophages *in vitro*, and this increases the growth of *M. tuberculosis* (Castaño et al., 2011). Based on previous studies investigating the roles of CD36 in *M. tuberculosis* infection and our experimental results, it was hypothesized that the up-regulation of CD36 in PBMCs is closely related to *M. tuberculosis* infection in patients with ATB. Nevertheless, low CD36 expression in the serum may promote the infection of *M. tuberculosis* further *in vivo*. This expression pattern of CD36 may be the common result of *M. tuberculosis* infection and resistance to *M. tuberculosis* infection.

*M. tuberculosis* infected macrophages from immunologically naïve guinea pigs increased the expression of CD36 *in vivo* (Palanisamy et al., 2012), and CD36 is increased in macrophages and mice infected by *M. tuberculosis* (Il’in and Shkurupy, 2019). In order to explore the roles of CD36 in the process of *M. tuberculosis* infection and the functions of exosomal CD36 in this infection process further, CD36 expression was knocked down using siRNA technology to analyze the interaction between *M. tuberculosis* and CD36. The results demonstrated that *M. tuberculosis* promoted CD36 expression on macrophages and in CD36-knockdown macrophages. Meanwhile, infection of *M. tuberculosis* down-regulated CD36 expression in the aforementioned cells. The opposite CD36 expression in macrophages and exosomes suggests that exosomal CD36 may serve critical roles in the process of *M. tuberculosis* infection.

The present study finally elucidated the profiles of exosome proteins in the peripheral blood of patients with ATB and analyzed the functions of significantly different proteins. The identified differentially expressed proteins associated with ATB infection, such as MHC-I and CD36, and LBP may be potential markers for the diagnosis of *M. tuberculosis* infection confirmed by ROC analysis in serum. However, their specific molecular mechanism in the process of *M. tuberculosis* infection needs to be studied further.

## Data Availability Statement

The original contributions presented in the study are included in the article/supplementary material, further inquiries can be directed to the corresponding author/s.

## Ethics Statement

The studies involving human participants were reviewed and approved by the Ethics Committee of Affiliated Kunshan Hospital of Jiangsu University. The patients/participants provided their written informed consent to participate in this study.

## Author Contributions

LT and JW conceived, designed, discussed the work, and revised the manuscript. MZ, YX, SL, and XY did the experiments. MZ, YX, and SL wrote and edited the manuscript. All authors read and approved the final version.

## Conflict of Interest

The authors declare that the research was conducted in the absence of any commercial or financial relationships that could be construed as a potential conflict of interest.

## Publisher’s Note

All claims expressed in this article are solely those of the authors and do not necessarily represent those of their affiliated organizations, or those of the publisher, the editors and the reviewers. Any product that may be evaluated in this article, or claim that may be made by its manufacturer, is not guaranteed or endorsed by the publisher.

## Footnotes

1
http://bonsai.hgc.jp/~mdehoon/software/cluster/software.htm

2
http://cello.life.nctu.edu.tw/

3
http://geneontology.org/

4
http://string.embl.de/

5
https://cytoscape.org/
